# Quality of Life in Polycystic Ovary Syndrome after Anti-Androgen Therapy using PCOSQOL-47 and PCOSQOL-42

**DOI:** 10.2174/0118715303376615251210134139

**Published:** 2026-03-17

**Authors:** Samih Abed Odhaib, Mahmood Thamer Altemimi, Adel Gassab Mohammed, Dheyaa Kadhim Al-Waeli, Miaad Jassim Mohammed, Haider Ayad Alidrisi, Husam Jihad Imran, Abbas Ali Mansour

**Affiliations:** 1 Department of Endocrinology, Thi-Qar Specialized Diabetes, Endocrine and Metabolism Center, College of Medicine, University of Sumer, Thi-Qar Health Directorate, Nasiriyah, Thi-Qar, Iraq;; 2 Department of Medicine and Endocrinology, Thi-Qar Specialized Diabetes, Endocrine and Metabolism Center, College of Medicine, University of Thi-Qar, Thi-Qar Health Directorate, Nasiriyah, Thi-Qar, Iraq;; 3 CABM-Endocrine, Adult Endocrinologist, Thi-Qar Specialized Diabetes, Endocrine and Metabolism Center, College of Medicine, University of Thi-Qar, Nasiriyah, Thi-Qar, Iraq;; 4 Department of Radiology, College of Medicine, Al-Refaee General Hospital, University of Sumer, Thi-Qar Health Directorate, Al-Refaee, Iraq;; 5 Department of Medicine and Endocrinology, Faiha Specialized Diabetes, Endocrine and Metabolism Center, Basrah Health Directorate, University of Basrah, Basrah, Iraq;; 6 Department of Medicine and Endocrinology, Misan Specialized Diabetes and Endocrine Center, Misan Health Directorate, Amarah, Misan, Iraq;; 7 Department of Medicine and Endocrinology, Faiha Specialized Diabetes, Endocrine and Metabolism Center, Basrah Health Directorate, University of Basrah, Basrah, Iraq

**Keywords:** HRQOL scales, anti-androgen therapy, questionnaires, quality of life, polycystic ovary syndrome, hyperandrogenism

## Abstract

**Introduction:**

Hyperandrogenism in polycystic ovary syndrome (PCOS) negatively affects health-related quality of life (HRQOL). Anti-androgens may improve both biochemical and patient-related outcomes.

**Objectives:**

To evaluate the impact of combined finasteride and spironolactone on HRQOL in married and unmarried women with PCOS using PCOSQOL-47 and PCOSQOL-42, respectively.

**Methods:**

A total of 263 married and 236 unmarried women with PCOS were enrolled from two tertiary centers. All received finasteride (5 mg) and spironolactone (100 mg) daily and were followed every four months over a year. HRQOL was assessed using PCOSQOL-47 for married and PCOSQOL-42 for unmarried women, alongside evaluating their biochemical and clinical hyperandrogenism.

**Results:**

After one year, significant reductions were observed in total and free testosterone, dehydroepiandrosterone sulfate, body mass index (BMI), and a decrease in modified Ferriman-Gallwey (mFG) scores. Both groups showed marked HRQOL improvement, greater in unmarried women. In PCOSQOL-47, psychological and emotional domains improved most, while fertility and sexual domains improved least. In PCOSQOL-42, menstrual and fertility domains had the highest gains, while coping improved least. Both groups had a 25% reduction in mFG score and 5% reduction in BMI.

**Discussion:**

This is the first study to evaluate combined finasteride and spironolactone on HRQOL domains in PCOS using PCOSQOL-47 and PCOSQOL-42. Prior studies assessing anti-androgens used other tools, without distinction between married and unmarried women.

**Conclusion:**

Combined finasteride and spironolactone significantly improved all HRQOL scales and reduced hyperandrogenism within the first four months in women with PCOS, supporting its role alongside lifestyle measures.

## 1 INTRODUCTION

Polycystic ovary syndrome (PCOS) is a multifaceted endocrine disorder affecting women of reproductive age, characterized by hyperandrogenism, menstrual irregularities, and polycystic ovarian morphology [1]. Among the pharmacological interventions aimed at managing the symptoms of PCOS, anti-androgens are crucial in mitigating the effects of hyperandrogenism, thereby improving the health-related quality of life (HRQOL) [2]. Spironolactone and finasteride are two such anti-androgens that have garnered attention for their efficacy in treating hirsutism, acne, and alopecia in women with PCOS [3].

Spironolactone, a potassium-sparing diuretic with antiandrogenic properties, is frequently prescribed off-label for the management of hyperandrogenic symptoms in women with PCOS [4-6]. By competitively inhibiting androgen binding to its receptor and reducing androgen synthesis, spironolactone effectively ameliorates hirsutism and acne, common concerns among women with PCOS [7]. Moreover, spironolactone’s diuretic effect may offer additional benefits by alleviating symptoms of fluid retention and bloating, potentially enhancing the overall QOL for affected individuals [5].

Finasteride, a 5-α-reductase inhibitor, blocks the conversion of testosterone to its more potent metabolite, dihydrotestosterone (DHT) [8]. While primarily indicated for the treatment of male-pattern baldness, finasteride has also been investigated for its efficacy in managing hirsutism and alopecia in women with PCOS [4]. By reducing DHT levels, finasteride can slow the progression of hair loss and decrease hirsutism, thereby improving cosmetic concerns and enhancing the psychosocial well-being of affected individuals [9].

Spironolactone and finasteride represent important therapeutic options in the management of hyperandrogenism-associated symptoms of PCOS. By targeting different aspects of androgen excess, these anti-androgens offer complementary mechanisms of action. They may be used alone or in combination to optimize clinical outcomes and improve the QOL for affected individuals [3].

This study aims to evaluate the effect of combination anti-androgen therapy (finasteride and spironolactone) on the different domains of HRQOL of married and unmarried women with PCOS using PCOSQOL-47 and PCOSQOL-42 questionnaires, respectively.

## 2 MATERIALS AND METHODS

After completing the validation phase of the PCOSQOL-47 and PCOSQOL-42 in August 2020 [10, 11], this a cross-sectional study conducted on the married and unmarried women diagnosed with PCOS. Sample size was calculated according to equation (1) [12]:


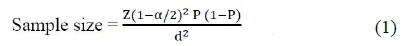


Here, Z_1-a/2_ is a standard normal variate at 5% type 1 error (*P*<0.05); it is 1.96, as this study considered the level of significance at 0.05. P = proportion of PCOS in the population, which was (10%) according to a systematic review and meta-analysis [13]. d = Absolute error or precision, and the precision/absolute error of 5% and at a type 1 error of 5%. The smallest sample size necessary to do this study was 139, but the real number of participants in this study was 406 married and 362 unmarried women for more satisfaction.

### 2.1 Inclusion/Exclusions

A total of 263 married and 236 unmarried women diagnosed with PCOS according to Rotterdam criteria were included from two tertiary centers Thi Qar and Faiha Specialized Diabetes Endocrine and Metabolism Centers) in Thi Qar and Basrah (TDEMC and FDEMC). All received finasteride (5mg) and spironolactone (100mg) daily and were followed every four months over a year. HRQOL was assessed using PCOSQOL-47 for married and PCOSQOL-42 for unmarried women, alongside evaluating their biochemical and clinical hyperandrogenism.

Any woman who did not meet these criteria, or was treated by either contraceptive pills or other antiandrogen therapy, lost follow-up, planned to get pregnant, or was unwilling to participate, was excluded, as shown in Fig. (****1****).

We provided detailed descriptions of all available therapeutic options offered by the medical team at Thi Qar and Faiha Specialized Diabetes Endocrine and Metabolism Centers in Thi Qar and Basrah (TDEMC and FDEMC), respectively. These options comprise:

- Option I: Administration of a finasteride tablet 5 mg once daily and a spironolactone tablet 100 mg once daily, in conjunction with self-administered or specialized dermatological hair removal.
- Option II: Additional treatment with oral contraceptive pills (OCP).
- Option III: Other anti-androgen therapy other than finasteride and spironolactone.

Employing a patient-centric, symptom-driven approach, the medical teams at both centers thoroughly deliberated each woman's treatment options, including a full description of the treatment course and possible side effects. Then, a healthcare team made a treatment decision for each woman individually.

**Fig. (1):**
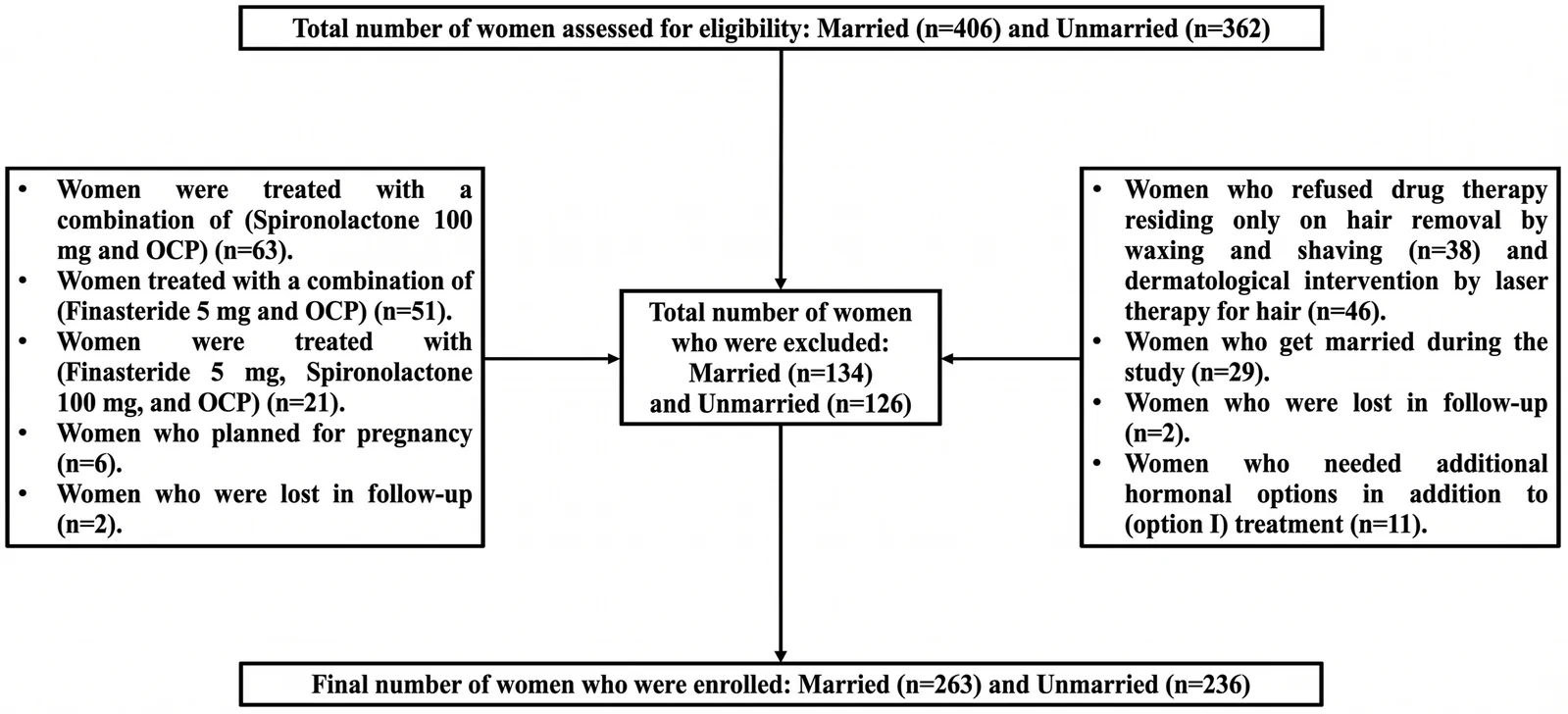
The consort-style flow diagram of women with polycystic ovary syndrome to illustrate participants’ recruitment, attrition, and completion.

The 406 married women enrolled during the validation phase were also included in this study phase. Only 263 women who continued (Option I) treatment were enrolled, and their data were entered in the final analysis (Fig. **1**).

Similarly, the 362 unmarried women initially enrolled during the validation phase were also included in this study. Only 236 unmarried women who continued (Option I) treatment were enrolled, and their data were entered in the final analysis (Fig. **1**).

All women in both cohorts were scheduled for quarterly visits to the tertiary centers, during which signs and symptoms were assessed, and the PCOSQOL-47 and PCOSQOL-42 were administered as appropriate. A medical team member assisted any woman wishing to complete the questionnaire, ensuring that women could respond to or decline any item in any domain.

All women were instructed to bring their empty anti-androgen drug sheets (finasteride and spironolactone) to confirm compliance with the recommended anti-androgen regimens. Women were advised to notify healthcare providers of any negative effects they experienced throughout the treatment period at their scheduled appointments.

General characteristics of women, including age, age at menarche, age at PCOS diagnosis, PCOS duration, history of menstrual irregularity, and acne, were recorded. Additional parameters such as age at marriage, age at first pregnancy, number of pregnancies, and history of abortion were registered for married women only.

### 2.2 Clinical Evaluation

Standard anthropometric measurements were conducted. Height was measured using a seca^®^217 mobile stadiometer (Seca, Chino, CA, USA), and weight was measured using the seca^®^763 electronic weight station (Seca). Body mass index (BMI) was calculated as weight in kilograms divided by height in square meters. A BMI < 25 kg/m^2^ was considered normal, while ≥ 25 kg/m^2^ was considered overweight/obese.

Hirsutism was clinically and visually assessed using the semi-objective modified Ferriman-Gallwey (mFG) score interpretation at each visit. This system evaluates 9 body sites (upper lip, chin, chest, upper and lower back, upper and lower abdomen, upper arm, forearm, thigh, and lower leg), with each site rated from 0 (no hair) to 4 (frankly virile). To decrease this variability in testing, we requested patients not to use laser or electrolysis for at least three months, not to depilate or wax for at least four weeks, and to avoid shaving for at least five days before the examination. The evaluation was done by the same examiner in each center to decrease the inter-observer variability. Hirsutism severity was graded as mild (mFG score ≤ 15), moderate (mFG score 16-25), severe (mFG score > 25), or absent (mFG score ≤ 8) [4, 14, 15].

The severity of female pattern hair loss (FPHL) was evaluated periodically using Sinclair’s scale, which categorizes it as minimal (Grade 1), moderate (Grades 2-4), or severe (Grade 5) [16]. The results were not included in the final results as this scale is subjective, depending on the visual analog score, and lacks objectivity.

#### 2.2.1 Using PCOSQOL-47 and PCOSQOL-42: Score Calculation in a Cohort

As outlined in Odhaib *et al*. [11], each item response was recorded using a five-point Likert scale:

- Never = 5 (no effect on HRQOL).
- Seldom = 4.
- Quite often = 3.
- Very often = 2.
- Always = 1 (maximal effect on HRQOL).

Women recorded their responses to various items across different domains of the questionnaires based on how they perceived these items affected their HRQOL. The total points for each item within a domain were divided by the number of items scored to calculate the final domain score, presented as (mean ± standard deviation).

The overall score for each questionnaire was calculated by dividing the sum of individual domain scores by the number of domains assessed.

The interpretation of domain score points or final questionnaire scores was classified as follows:

- Scores in the first interval (1 to < 3 points) indicated significant effects on HRQOL.
- Scores in the second interval (3 to < 4 points) suggested a moderate effect on HRQOL.
- Scores in the third interval (4 to < 5 points) indicated a minimal effect on HRQOL.
- A score of 5 points indicated no effect on HRQOL.

The overall score for each questionnaire every four months was calculated by summing all domains and dividing by 5.

### 2.3 Laboratory Testing

An early morning venous sample was collected after at least eight hours of overnight fasting. Ten milliliters of venous blood were collected in a gel tube and centrifuged at 4100 xg by NUVE-NF 800.

Total testosterone (TT), SHBG, dehydroepiandrosterone sulfate (DHEA-S), and insulin levels were assessed using electrochemiluminescence by Roche Cobas e411 Analyzer (Germany) with standardized mean values. Calculation of free testosterone (FT) was established by Vermeulen *et al*. [17].

Fasting plasma glucose (FPG) was measured using Cobas 311 (Roche Diagnostics-Mannheim-Germany). Insulin resistance (IR) was assessed using the Homeostasis- Model Assessment of IR (HOMA-IR) formula, with a value of 2.11 or more considered diagnostic for IR [18].

### 2.4 The Lifestyle Intervention

The caring team provided preliminary information about weight loss and a healthy diet. This part was not standardized because of the individual variations regarding food preferences, home conditions, and personal motivations. The inability to recall daily food items makes it very difficult to register by women and the caring team.

### 2.5 Statistical Analyses

Data were analyzed using IBM SPSS Statistics for Windows, version 26.0 (IBM Corp., Armonk, NY). Data were expressed as (mean ± standard deviation) or frequency %. One-way Analysis of Variance (ANOVA) was used to compare means. A two-tailed *p*-value ≤ 0.05 was considered statistically significant at a 95% confidence interval. Repeated Measures Statistics, Pairwise Comparison, age as a covariate, and Estimated Marginal Means with Bonferroni correction were utilized for detailed visual estimation using line graphs to illustrate scores of different domains within both questionnaires during the four visits. A paired sample t-test was used to compare levels of some biochemical and physical parameters before and after anti-androgen therapy.

## 3 RESULTS

Table **1** shows the general characteristics of women in both cohorts. While married women have a higher mean age than unmarried women, their age ranges are similar. Both cohorts were diagnosed with PCOS during late adolescence, which is typical for this condition. The range of pregnancies (0 - 8) is wide, likely influenced by the quarter of married women with a history of abortion. Despite the age difference favoring the married cohort, married women have nearly double the PCOS duration of unmarried women, though the duration ranges are similar.

Approximately 80% of women in both cohorts had mean weights and BMIs in the overweight and obese ranges, which is common among PCOS patients.

Unfortunately, all women in either cohort had a certain degree of hirsutism, and around 93% of enrolled women in both cohorts had mild to moderate hirsutism, reflected by high mFG scores around 16 points, while the remainder had severe forms.

FPHL was absent in around 30% of married women (n=80) and 19% of unmarried women (n=45), with the rest showing varying degrees of hair loss reflected by unacceptable Sinclair’s scores.

Menstrual irregularity was more prevalent in the married cohort (91%, n=239) compared to the unmarried cohort (78%, n=184).

Although subjective, acne at presentation was observed in about half of married women and a quarter of unmarried women.

In Table **2**, the testosterone, DHEA-S, and HOMA-IR levels at enrollment compared to their values at the end of the study. At enrollment, approximately a third of women in both cohorts had high (measured) TT levels, with more than half having their calculated FT above the ULN. Yet, the TT and FT means were in the acceptable normal or slightly above the ULN. The testosterone level in both cohorts improved, with only 2% of the married women (n=5) having their TT > ULN, while all unmarried women had their TT in the normal reference ranges.

Based on reference values, about a quarter of women in either cohort had high DHEA-S levels. The level of DHEA-S in both cohorts was improved, with only 2% of the married women (n=5) having their DHEA-S > ULN, while all unmarried women had their DHEA-S in the normal reference ranges.

Dramatic changes were seen in the HOMA-IR levels during the study. Around 60% of women in both cohorts exhibited signs of IR, which high HOMA-IR reflected. After one year, 18% of married women (n=47) still had high HOMA-IR, compared to 25% of unmarried women (n=59).

The decline in the TT, FT, DHEA-S, and HOMA-IR in either cohort was assessed using the (Paired Sample t-test) in Table **3**. It showed a significant decline in these parameters across the year of the study on anti-androgen therapy to control the hyperandrogenic signs and symptoms of PCOS.

We used repeated measures statistics and line graphs to assess the extent of this significant reduction and its relation to HRQOL in both cohorts.

### 3.1 Results of PCOSQOL-47 (Married Women with PCOS)

Over the 12-month study period, married women with PCOS experienced a significant improvement in their HRQOL, evident in the PCOSQOL-47 scores, shifting from marked to marginal effects for many women. This improvement began within the initial four months and persisted throughout the study, as depicted in Fig. (**2**). Subgroup analysis further confirmed the significance of this change across visits.

Table **4** outlines detailed changes in domain effects for married women with PCOS, comparing enrollment to end-of-year scores:

(1) Psychological and Emotional Status Domain: All women reported significant HRQOL improvement in this domain, transitioning from initial to higher-ranked effects.

(2) Fertility and Sexual Life Domain: While the response was minimal, significant changes occurred, with more women reporting marginal effects by the study's end.

(3) Body Image Domain: Although 33% of women reported no change, others experienced varying degrees of significant improvement.

(4) Hair Disorders and Acne Domain: While 35% saw no change, others showed better results.

(5) Obesity and Menstrual Disorders Domain: While 21% saw no change, a few experienced worsened HRQOL.

Improvement order in descending fashion in PCOSQOL-47 domains was (Psychological and Emotional Status), (Obesity and Menstrual Disorders), (Hair Disorders and Acne), (Body Image), and lastly (Fertility and Sexual Life) domain.

Overall, 85% of women showed significant HRQOL improvement, with 14% not improving and a few worsening. Fig. (**3**) panels Panel (A): (Psychological and Emotional Status Domain). Panel (B): (Fertility and Sexual Life Domain). Panel (C): (Body Image Domain). Panel (D): (Hair Disorders and Acne Domain). Panel (E): (Obesity and Menstrual Disorders Domain), illustrates domain-specific changes, showing significant score shifts towards better results.

Additionally, there was a minimal yet significant BMI change during the study, remaining in the obese and overweight ranges. This change began early and persisted throughout the year.

**Fig. (4):**
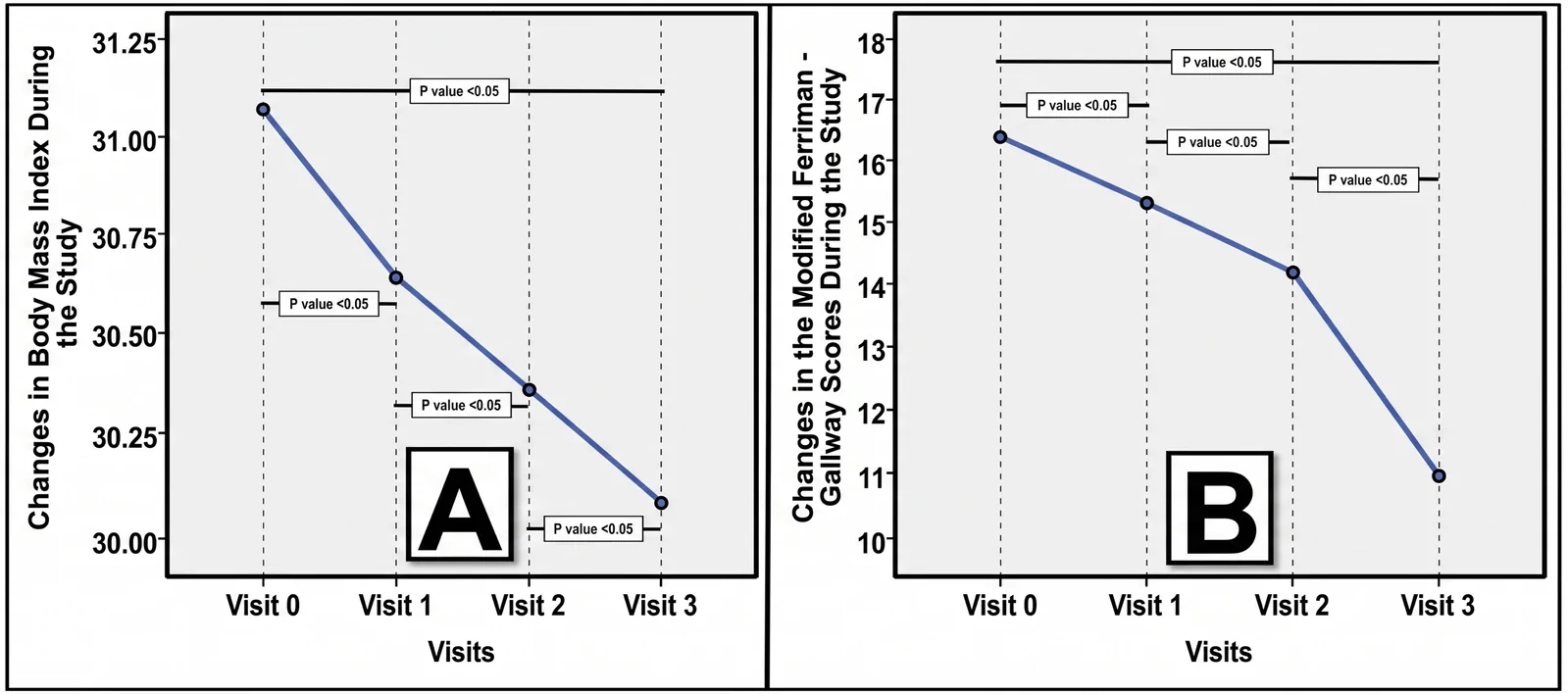
Panel (**A**): The changes in the body mass index for married women with polycystic ovary syndrome across the study with the subgroup analysis using Repeated Measures Statistics. Panel (**B**): The changes in modified Ferriman – Gallwey Score for married women with polycystic ovary syndrome across the study with the subgroup analysis using Repeated Measures Statistics.

The percentage of reduction in weight for the 263 married women was (-2.72 ± 0.32) %, while it was more if we consider the subgroup analysis of the 151 married women with BMI in the obesity range, which was (-4.64 ± .47%) (Fig. **4A**). Furthermore, women experienced a marked decline in mean mFG score throughout the study, starting early and remaining significant across visits despite lacking specialized local therapy for hirsutism (Fig. **4B**). The mean percentage of mFG reduction for married women reached around 25 ± 1% with a mean reduction of 5 ± 1 points.

### 3.2 Results of PCOSQOL-42 (Unmarried Women with PCOS)

Over the 12-month study period, unmarried women with PCOS experienced a significant improvement in their HRQOL, as reflected in the overall PCOSQOL-42 scores, transitioning from marked to minimal effects for the majority. This improvement commenced within the initial four months and persisted throughout the study, as illustrated in Fig. (**5**). The change was notable across the study and during subgroup analyses between visits.

Table **5** details the robust changes in domain effects for unmarried women with PCOS, comparing enrollment to end-of-year scores. Across domains:

(1) Psychological and Emotional Status Domain: Nearly all women (97%) showed significant HRQOL improvement, with only a few not improving.

(2) Menstrual Disorders and Fertility Domain: The majority (89%) demonstrated improvement, though some experienced deterioration.

(3) Body Image Domain: Virtually all women (99%) showed improvement.

(4) Hair Disorders and Acne Domain: Most women (84%) experienced significant improvement, with no deterioration.

(5) Coping Domain: A significant majority (77%) showed improved coping abilities.

Improvement order in PCOSQOL-42 domains in descending fashion was (Menstrual Disorders and Fertility), (Body Image), (Psychological and Emotional Status), (Hair Disorders and Acne), with the (Coping Domain) having the lowest change.

Overall, there was a significant improvement in HRQOL for most unmarried women with PCOS, with no deterioration in the net effect. Fig. (****6****) panels illustrate domain-specific changes. Panel (A): (Psychological and Emotional Status Domain). Panel (B): (Menstrual Disorders and Fertility Domain). Panel (C): (Body Image Domain). Panel (D): (Hair Disorders and Acne Domain). Panel (E): (Coping Domain), all showing significant score shifts except for the hair disorders and acne domain, which remained in the highest values of the marginal effect region.

Like the married women cohort, there was a significant yet minimal reduction in BMI among unmarried women, remaining in the obese and overweight range.

**Fig. (7):**
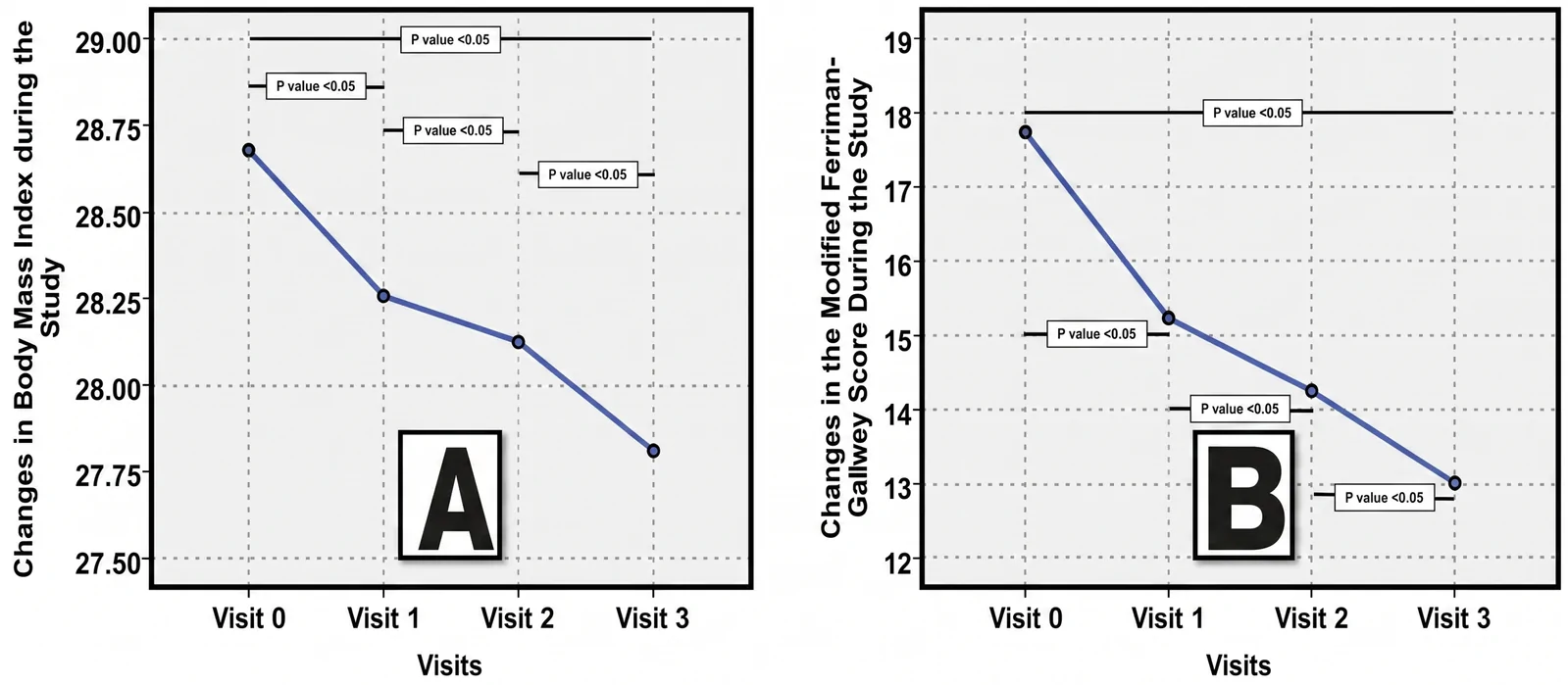
Panel (**A**): The changes in the body mass index for unmarried women with polycystic ovary syndrome across the study with the subgroup analysis using Repeated Measures Statistics. Panel (**B**): The changes in modified Ferriman – Gallwey Score for unmarried women with polycystic ovary syndrome across the study with the subgroup analysis using repeated measures statistics.

The weight reduction percentage for the 236 unmarried women was (-2.04 ± 0.43%). In comparison, it was more if we consider the subgroup analysis of the 93 unmarried women with BMI in the obesity range, which was (-6.57 ± 0.47%) (Fig. **7A**).

Additionally, there was a marked decline in overall mFG score across the study year, starting early and remaining significant across visits, despite lacking specialized local therapy for hirsutism (Fig. **7B**). The mFG reduction percentage was (26 ± 1%), with a mean reduction of (5 ± 1) points.

Table **6** summarized comparison of domain-specific HRQOL improvements between married and unmarried women with PCOS after one year of anti-androgen therapy. It provides the inclusion of subgroup comparisons and domain-specific outcomes provides greater insight into differences between married and unmarried women.

## 4 DISCUSSION

PCOS is a common chronic condition affecting women of reproductive age. Due to limited data, assessing QOL during follow-up is crucial in understanding disease progression. Treatment requires a holistic approach, including medication, cosmetic interventions, and psychological support tailored to individual preferences.

This is the first study to evaluate the effect of a combination of anti-androgens (finasteride and spironolactone) on different HRQOL domains using PCOSQOL-47 and PCOSQOL-42. The studies that used this anti-androgen combination in women with PCOS or clinical hyperandrogenism used different tools to assess their HRQOL, regardless of whether the women were married or not, *i.e.*, sexually active or not.

Table **1** provides a clue about the similarity between the two cohorts in their general characteristics, with some minor differences, especially in the extent and severity of signs of clinical hyperandrogenism, like hirsutism score and FPHL.

Treatment with an antiandrogenic drug is off-label yet widely used [2, 3, 14]. This anti-androgen combination (spironolactone and finasteride) works in different dimensions of PCOS.

Tables **2** and **3** show a significant, drastic reduction in hyperandrogenism and IR parameters in women with PCOS during treatment with anti-androgens (finasteride and spironolactone). At the start of the study, the mean values of TT, FT, and DHEA-S concentrations were within normal ranges, but a significant percentage were above normal. However, HOMA-IR indicated IR in about two-thirds of the participants. After one year of treatment, the means of these parameters and percentages improved, reflecting better HRQOL effects. The study found that the treatment with anti-androgens significantly improved the chemical parameters of hyperandrogenism and IR. The concentrations of certain parameters decreased after one year of treatment, leading to improvements in HRQOL.

During the 12-month study period, there was a significant improvement in all domains of the (PCOSQOL-47 and PCOSQOL-42) for married and unmarried women with PCOS, respectively, with better results for unmarried women who had the best improvement of their HRQOL compared to the married women, which may be related mainly to the collective changes regarding body image, menstruation, fertility, and sexual life.

The menstrual disorders and fertility domain showed the greatest improvement for unmarried women using PCOSQOL-42, while the psychological and emotional status domain showed the greatest improvement for married women using PCOSQOL-47. The improvement in PCOSQOL-47 and PCOSQOL-42 is affected by the ranking of the domains within the questionnaires (Table **6**). The improvement in the dysmorphic features or bodily dissatisfaction, weight, menstrual problems, and fertility issues was evident through the final score of both questionnaires, although the exact mechanism is unknown. The causal relation between the use of anti-androgen drugs and lifestyle intervention cannot be assured in this study due to the complex pathogenicity of PCOS, and is not merely related to excess androgen and weight only. Still, the items from both questionnaires may be perceived differently by women in both cohorts, especially from a sexual point of view. The additional effects of previous pregnancies and abortion may be related, although we could not measure their effect on unmarried women in our community.

PCOSQOL-47 showed that 85% of women showed significant HRQOL improvement, with 14% not improving and about 1% showing worsened HRQOL. In contrast, PCOSQOL-42 showed a significant improvement in HRQOL for most unmarried women with PCOS, with no deterioration in the net effect. Remember that the total effect denoted in Tables **4** and **5** is not the summation of the effects in the domains. The final effect is calculated for the whole domain as one entity, as illustrated in the methods section.

Notably, the significant improvement in the PCOSQOL-47 and PCOSQOL-42 started early in the first four months in all domains, as seen in the repeated measures statistics in different panels of Figs. (**2** and **5**). The weight reduction and anti-androgen combination intervention significantly changes the HRQOL of those women. They did not necessarily improve their grade in the domain; women were better in the same grade. The final results in PCOSQOL-47 and PCOSQOL-42 showed higher grades than their initial reported grades. The anti-androgens (finasteride and spironolactone) may produce earlier changes within the first four months and maintain this effect during the year of therapy. Yet, even with this earlier start of action within four months, the one year of therapy was not enough to produce the optimal or ethnically acceptable mFG score in either cohort, although the reduction in mFG reached 25%.

While the anti-androgen effect of finasteride and spironolactone and their mechanisms of action allude to plausible benefits, the 2023 PCOS guidelines [1] concluded that evidence was lacking for using all identified anti-androgen medications in PCOS. Existing systematic reviews have focused on women with idiopathic hirsutism or broader hirsute populations. There are significant differences in how these two anti-androgen drugs work, even though they both fall into the same category. Many studies have established that both spironolactone and finasteride have beneficial effects in managing women with hirsutism [19-22].

The anti-androgen action of finasteride and spironolactone can be quantified clinically by the percentage reduction in the mFG score from the baseline at the study end, which reached about 25% by reducing about five points from the overall mFG score in both cohorts. The resultant changes in the parameters of chemical hyperandrogenism were significant using this combination for a one-year duration, even in women with normal testosterone levels. A similar and better reduction was found by Keleşimur *et al*. (2004) [23], and Unlühizarci *et al*. (2002) [24] on a similar cohort of Turkish women who had PCOS and were ethnically like Iraqi women, *i.e.*, both are Middle Eastern with similar reference ranges for the mFG scores. A previous systematic review [25] assessed 26 controlled clinical trials using various anti-androgens and found that spironolactone 100 mg/d led to an average 38.4% improvement in the mFG score. The initial response during the (finasteride and spironolactone) was noticed as early as four months, yet it did not reach optimal levels of mFG scores at the study end.

As most anti-androgen combination treatments need to be continued for 6 - 12 months before an effect can be observed, all women receive the appropriate consultation about all aspects of therapy at each visit to prevent disappointment and are encouraged to use interim cosmetic measures as recommended by other studies [23, 24, 26, 27]. These measures facilitate the reduction in the perceived effect of parameters of clinical hyperandrogenism, and the effects persist after their withdrawal in most patients, with no possible explanation of this finding given the complexity of the pathogenicity of PCOS and the broad antiandrogenic activity of finasteride and spironolactone.

Hirsutism can lead to psychological distress, low self-esteem, negative self-image, feelings of shame and embarrassment, social disabilities, and even depression, with a significant impact on QOL [28-31]. Hair growth cycles vary for different body parts, and therefore, treatment for at least 6 - 12 months is essential for optimal effect, but continuous treatment may be required [15].

The guidelines expected a decline in the mFG score by 15% within six months after the start of therapy, although there is considerable variation among individual women. The maximum effect is 9 to 12 months [7]. The effect is more attributed to the spironolactone, with less effect attributed to the 5α-reductase inhibitor finasteride as an anti-androgen. Tartagni *et al*. study used finasteride to treat hirsutism in adolescent girls. It showed a minimal reduction in the mFG score with no difference in the parameters of chemical hyperandrogenism or BMI [32].

While hirsutism indicates excessive androgen activity at the pilosebaceous unit, its severity correlates weakly with the extent of androgen excess [1, 3, 11, 33]. This seeming contradiction suggests that hirsutism not only mirrors circulating androgen concentrations but also depends on peripheral androgen metabolism, tissue sensitivity to androgens, and other hormonal factors such as insulin resistance and compensatory hyperinsulinism [15].

These complex mechanisms of action can explain the normal testosterone values found during spironolactone treatment in women: the block of androgen receptors could increase luteinizing hormones and androgens. Meanwhile, the direct effect at the ovarian level and the stimulation of aromatase could reduce testosterone synthesis [5].

The present study shows a decline in the age-specific DHEA-S even from its original normal ranges, while other studies did not show such changes with the same dose combination [22, 23].

Our results did not include serial Sinclair's score for any women except before therapy due to its total subjectivity, operator bias, and relative changes compared to the mFG score, which is semi-objective. There was no trichoscopy or assessment of the exact hair thickness and distribution in a unit area.

The FPHL affects various age groups, triggering significant negative psychosocial and physical impacts, including heightened distress over hair loss, negative body image, lower self-esteem, and diminished HRQOL [11, 16, 34]. Personal perceptions of hair loss severity closely align with its overall impact on HRQOL [11, 35]. Adolescent girls also suffer from these psychosocial effects, experiencing impaired functioning in daily life and relationships [11, 36]. That is why any minor improvement in hair-related items could significantly affect the overall scores of PCOSQOL-47 and PCOSQOL-42.

Still, the evidence may be conflicting and complex. The 5α-reductase activity is increased in the scalp of women with clinical hyperandrogenism, and this probably contributes to the pathogenesis of hair loss [8], leading to excessive DHT production in hair follicles, causing hair miniaturization [37]. In a setting where circulating androgens are normal, scalp sensitivity to androgens is likely an important pathophysiology component [8].

The reason for (no response) or minimal effect response in different women may be due to the fact that type I more than type II 5α-reductase activity is increased in FPHL, which may be the consequence of increased circulating androgens [8, 38], rendering finasteride minimally effective in those women.

Another important and complex integrated pathophysiological relationship is between hyperandrogenism and IR because obesity, especially abdominal obesity, reduces SHBG levels and increases tissue-specific effects of androgens in the affected tissues [39, 40]. It also boosts testosterone and non-SHBG-bound androgen production like DHEA and androstenedione [40], underscoring weight management's impact on PCOS hormonal dynamics. Still, we could not establish any relation between spironolactone and the SHBG level, given that we used a combination therapy.

The mean values of weight, BMI, and HOMA-IR reduction at the study end were minimal yet significant in either cohort, or did not reach the normal reference ranges for the majority of women. The changes in HOMA-IR and weight were not attributed to the anti-androgen therapy but to the modest lifestyle modification that was advised by the caring team, which cannot be quantified. This study showed that women in either cohort maintained a weight reduction of around 5% of their initial weight at enrollment.

Our study, consistent with prior research associating body weight with dissatisfaction, affirms the substantial impact of excess body weight on both physical and mental well-being, emphasizing its influence on self-reported health status, fostering weight stigma, social isolation, and loneliness, impacting psychosocial adjustment, and heightened dysmorphic concerns [11, 41-45]. Modest weight loss, typically 5% to 10% of initial body weight, has significantly improved many features of PCOS [1, 3]. This includes enhancements in reproductive health, such as menstrual cyclicity and fertility, and metabolic improvements, such as reduced IR [1, 46-49].

Crucially, the improvement in HRQOL emphasizes how important it is to adopt a patient-centered approach to PCOS care rather than relying solely on biochemical normalization. Addressing dermatological symptoms and emotional distress is just as important for long-term adherence and satisfaction as traditional therapies like weight loss and metformin, especially in insulin-resistant phenotypes [44-47].

The study findings also suggest that any reduction in BMI and parameters of biochemical hyperandrogenism affects HRQOL through the influence of dysmorphic concerns. Women with PCOS are often recommended to make lifestyle changes to support weight management and overall health despite limited direct evidence [1, 3].

The present study provides clear evidence of how we can improve the QRQOL of women with PCOS using anti-androgen agents like finasteride and spironolactone. The baseline variations in HRQOL scores might have influenced the degree of improvement, particularly in the emotional and reproductive domains. This provides additional context for interpreting treatment outcomes (Table **6**).

Several limitations in this study were apparent. Being the first study to evaluate changes in PCOSQOL-47 and PCOSQOL-42 questionnaires, the literature we used to compare resided on different assessment scales of HRQOL. The lack of a control group and placebo blinding for comparison of the results is another limitation. Our centers did not have the equilibrium dialysis methods for the FT estimation and relied on the mathematical estimation method established by Vermeulen *et al*. [17]. Also, our centers lack serum androstenedione and DHT measurements.

The individual influence of finasteride and spironolactone could not be assessed, as they were used in combination from the start of the study. Due to the nature of the study, we could not evaluate the proposed synergistic effect of OCP. Limitations in periodic clinical assessment by the mFG score were addressed and partially solved to be evaluated by the same examiner each time, the variability and bias in visual scoring cannot be ruled out. We needed more objectivity in FPHL using Sinclair's score, with no objective assessment of hair growth using trichoscopy or computer-assisted methods. Lifestyle changes through weight loss were not standardized, and it was not easy to assess and tailor because of the different food preferences, scheduling, adherence, and commitment of women.

Potential recall and reporting biases could not be ruled out, despite the questionnaire focusing solely on experiences from the past two weeks. These biases might have affected the responses of the participants. Furthermore, all data were self-reported, introducing the possibility of inaccuracies. Additionally, since the study participants shared a similar ethnicity, caution is needed in generalizing the findings to other ethnic groups.

## CONCLUSION

At the start of the study, we found that women with PCOS, whether married or unmarried, were significantly affected in all areas assessed by the PCOSQOL-47 and PCOSQOL-42 scales.

The use of anti-androgen combinations, specifically finasteride and spironolactone, demonstrated promising results in improving all domains of the PCOSQOL-47 and PCOSQOL-42. This suggests a potential avenue for more effective PCOS management despite the need for further understanding of the underlying mechanisms.

The research highlights a key finding that even with normal androgen levels, women with PCOS may still exhibit signs of clinical hyperandrogenism. This underscores the importance of a comprehensive approach to treatment. Anti-androgen combinations alone cannot normalize hirsutism, FPHL, IR, and lower BMI. Integrated lifestyle interventions are crucial for achieving these goals.

During the anti-androgen combination study, testosterone levels, weight, HOMA-IR, and mFG levels significantly reduced, but the study did not confirm a causal relationship.

Improvement in various domains of PCOSQOL-47 and PCOSQOL-42 began within the first four months of the study. However, the improvement could have been faster and reached a satisfactory level if it had depended on the pharmacodynamics of the anti-androgens and possible reasons for the delayed onset of improvement. Less than 1% of married women with PCOS showed a lower PCOSQOL-47 points, with no worsening observed in the PCOSQOL-42 points.

Women with PCOS from both cohorts showed significant improvement in all domains during the progression of the study. Although the degree of improvement differed between married and unmarried women, improvement followed a significant pattern across visits, as indicated by repeated measures statistics and subgroup analyses. The domains related to body image and self-satisfaction showed the most positive results.

The lifestyle interventions recommended for women in weight control were beneficial but only partially sufficient after applying culturally adapted PCOSQOL instruments in assessing HRQOL among different marital and sociocultural subgroups.

## FUTURE RECOMMENDATIONS

Further rigorous randomized controlled trials are essential to provide more definitive evidence regarding the efficacy of individual anti-androgens in women with PCOS. Additional research priorities include large-scale, multi-center longitudinal studies to compare the effectiveness of various types of anti-androgens, doses, combinations, and treatment schedules. Establishing optimal methods for monitoring adverse events and evaluating how these interventions impact domains assessed by the PCOSQOL-47 and PCOSQOL-42 scales is also crucial.

Multi-component interventions that integrate pharmacological and psychosocial treatments, along with exercise, are promising approaches to address the multifaceted physical and mental health concerns of women with PCOS. These interventions can enhance mental health outcomes measured by the PCOSQOL-47 and PCOSQOL-42 scales.

Furthermore, given that these scales have not been widely utilized in languages other than English, there is a need for culturally adapted questionnaires that can be employed in similar societal contexts with appropriate modifications.

## Figures and Tables

**Fig. (2):**
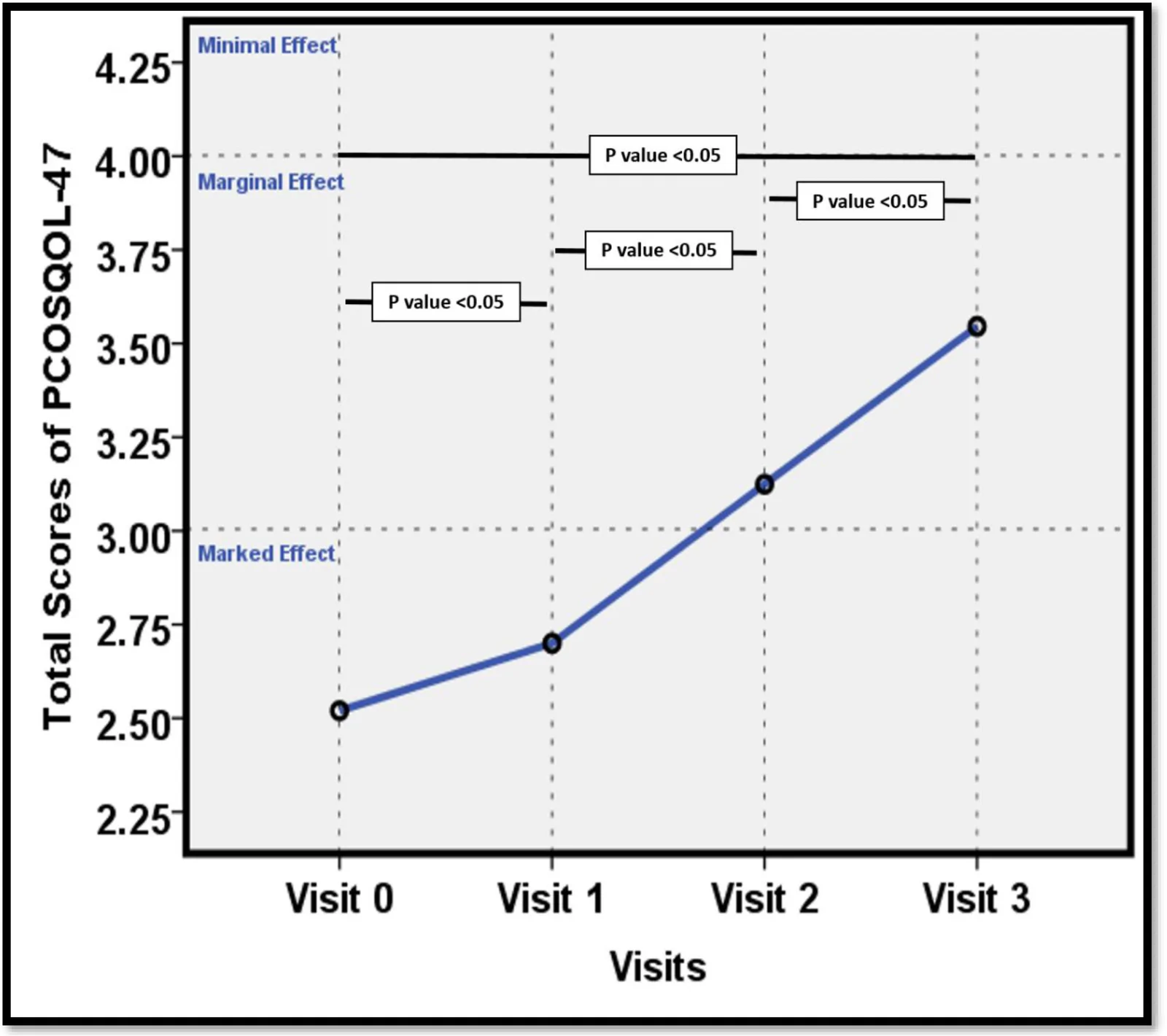
The PCOSQOL-47 overall results across the study with the subgroup analysis using repeated measures statistics.

**Fig. (3):**
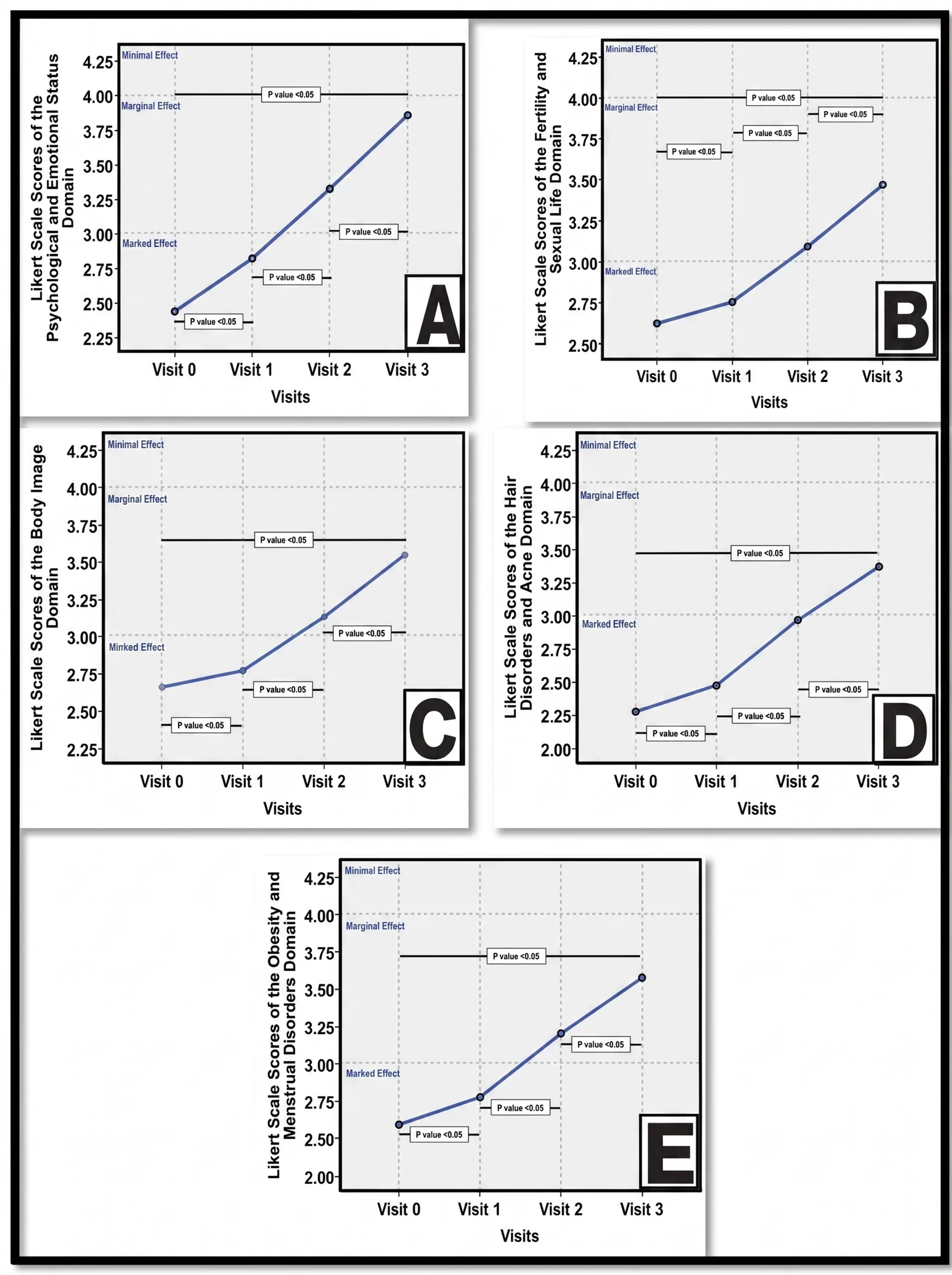
The detailed domain by domain score changes by PCOSQOL-47 across the study with the subgroup analysis using Repeated Measures Statistics. Panel (**A**): Psychological and Emotional Status Domain. Panel (**B**): Fertility and Sexual Life Domain. Panel (**C**): Body Image Domain. Panel (**D**): Hair Disorders and Acne Domain. Panel (**E**): Obesity and Menstrual Disorders Domain.

**Fig. (5):**
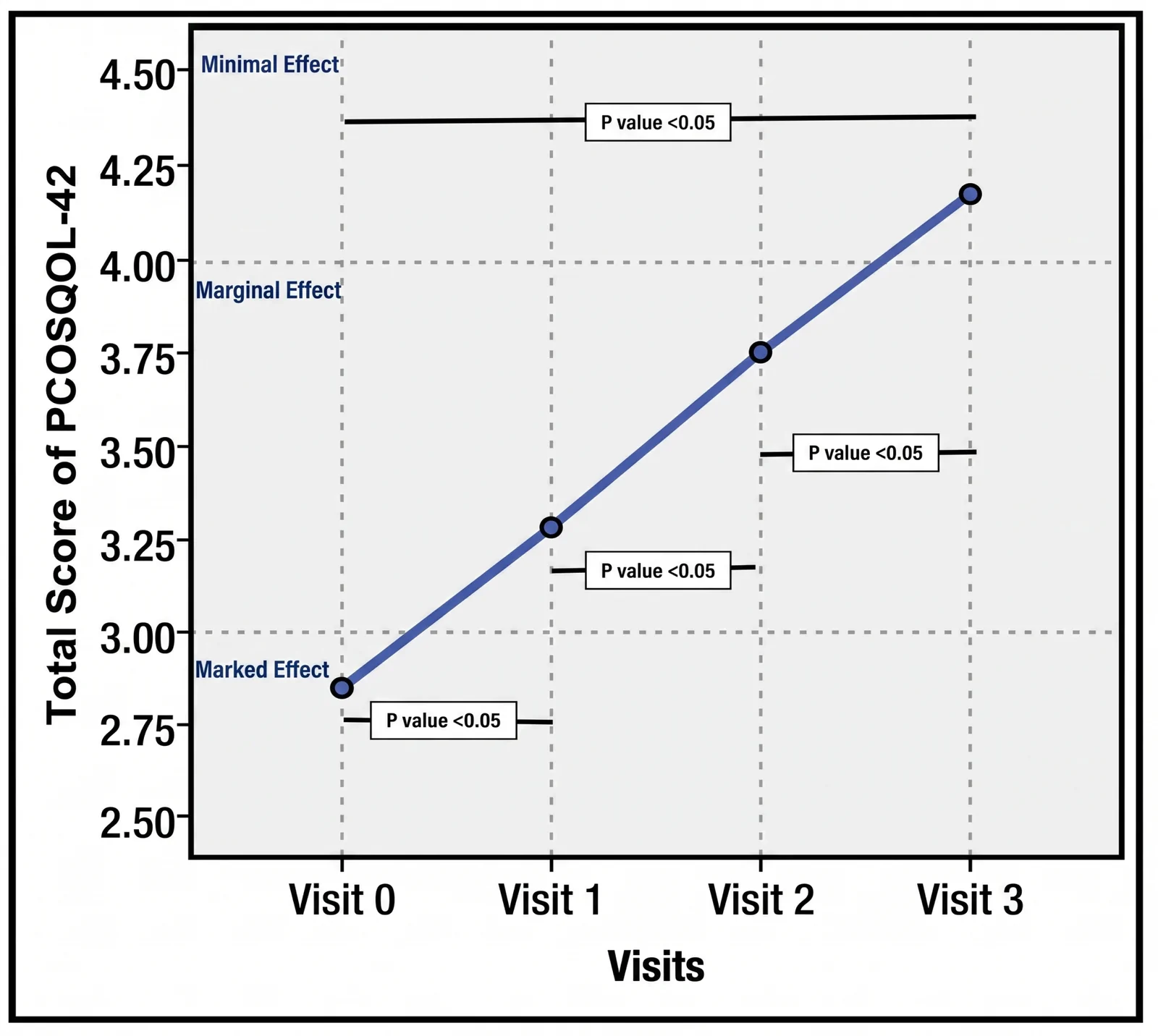
The PCOSQOL-42 overall results across the study with the subgroup analysis using Repeated Measures Statistics.

**Fig. (6):**
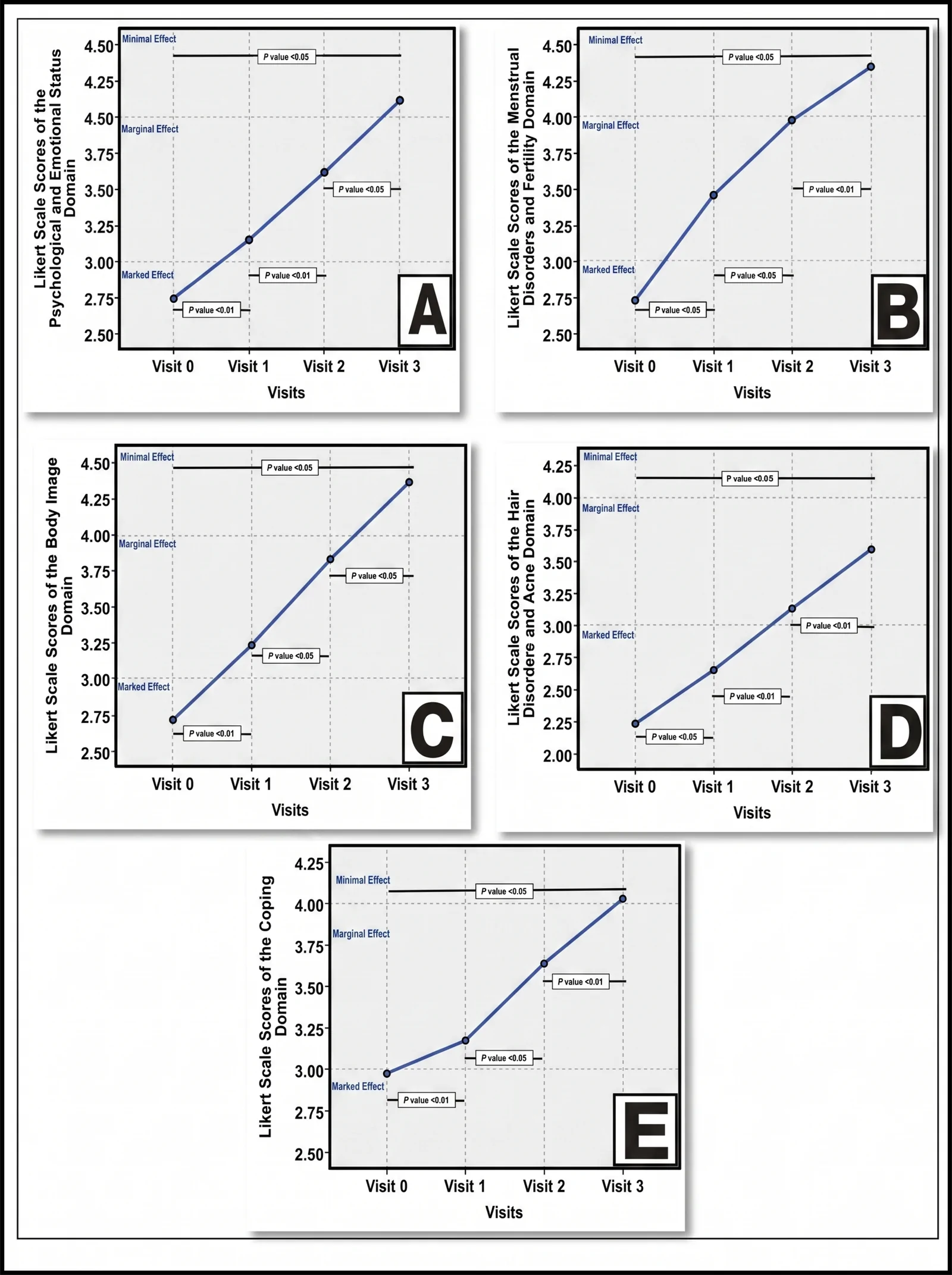
The detailed domain by domain score changes by PCOSQOL-42 across the study with the subgroup analysis using Repeated Measures Statistics. Panel (**A**): Psychological and Emotional Status Domain. Panel (**B**): Menstrual Disorders and Fertility Domain. Panel (**C**): Body Image Domain. Panel (**D**): Hair Disorders and Acne Domain. Panel (**E**): Coping Domain.

**Table 1:** Initial enrollment characteristics of the married and unmarried women with PCOS.

**Parameters**		**Married Women (n=263)**		**Unmarried Women (n=236)**	
		**Value**	**Range**	**Value**	**Range**
Age parameters Mean (SD) years	Age	29 (6)	17 – 39	23 (5)	15 - 46
	Age at diagnosis	17 (2)	15 - 31	19 (3)	14 - 35
	Age at menarche	13 (1)	11 - 15	13 (1)	11 - 15
	Age at marriage	21 (3)	16 - 32	-	-
	Age at first pregnancy	23 (4)	17 - 32	-	-
Number of pregnancies n (%)		2 (2)	0 - 8	-	-
Percentage of women with a history of abortion n (%)		63 (23.95)	-	-	-
Mean PCOS duration (SD)		11 (6)	1 - 23	4 (3)	1 – 29
Mean weight (SD) kg		79 (17)	46 - 135	71 (13)	46 - 120
Mean BMI (SD) kg/m^2^		31.16 (6.34)	19.43 – 52.73	28.72 (5.56)	19.78 – 48.44
BMI Categories n (%)	Overweight and obese	224 (85.2)	-	185 (78.4)	-
	Normal BMI	39 (14.8)	-	51 (21.6)	-
Mean mFG score (SD)		16 (5)	9 – 34	18 (5)	9 – 29
Hirsutism categories n (%)	Mild hirsutism	138 (52.47)	-	65 (27.54)	-
	Moderate hirsutism	113 (42.97)	-	153 (64.83)	-
	Severe Hirsutism	12 (4.56)	-	18 (7.63)	-
Percentage of women with FPHL n (%)		183 (69.6)	-	191 (80.9)	-
Mean Sinclair score (SD)		2 (1)	2 - 5	3 (1)	2 – 5
FPHL categories n (%)	Minimal	115 (43.7)	-	90 (38.14)	-
	Moderate	66 (25.1)	-	99 (41.95)	-
	Severe	2 (0.8)	-	2 (0.85)	-
	No FPHL	80 (30.4)	-	45 (19.06)	-
Percentage of women with menstrual irregularity n (%)		239 (90.9)	-	184 (78)	-
Percentage of women with acne n (%)		125 (47.5)	-	62 (26.3)	-

**Abbreviations:** BMI, Body Mass Index; FPHL, Female Pattern Hair Loss; mFG, Modified Ferriman-Gallwey; PCOS, Polycystic Ovary Syndrome; SD, Standard deviation.

**Table 2:** The testosterone, DHEA-S, and HOMA-IR levels at the enrollment compared to their values at the end of the study.

Parameters		Total Testosterone	Free Testosterone	DHEA-S	HOMA-IR
Married Women	Mean value at enrollment (SD)	37.02 (20.01)	0.67 (0.41)	262.45 (125.19)	3.92 (2.61)
	Percentage of women with more than ULN n (%)	80 (30.4)	151 (57.4)	61 (23.2)	169 (64.3)
	Mean value at end of study (SD)	18.71 (11.15)	0.33 (0.23)	114.32 (46.92)	2.25 (1.15)
	Percentage of women with more than ULN n (%)	5 (1.9)	35 (13.3)	5 (1.9)	47 (17.9)
Unmarried Women	Mean value at enrollment (SD)	40.92 (25.16)	0.75 (0.49)	246.41 (146.86)	3.85 (2.77)
	Percentage of women with more than ULN n (%)	87 (36.9)	128 (54.2)	57 (24.2)	142 (60.2)
	Mean value at end of study (SD)	23.11 (8.47)	0.46 (0.19)	179.19 (75.13)	2.90 (2.50)
	Percentage of women with more than ULN n (%)	0 (0)	54 (22.9)	0 (0)	59 (25.0)

**Abbreviations:** DHEA-S, Dehydroepiandrosterone Sulfate; HOMA-IR, Homeostatic Model Assessment for Insulin Resistance; SD, Standard Deviation; ULN, Upper Limits of Normal.

**Table 3:** Paired sample t-test for the changes in testosterone, DHEA-S, HOMA-IR, and BMI levels at the end of a year of the study.

Change in Parameters over a Year		Mean	SE Mean	95% C.I. of the Difference		*p*-value
				Lower	Upper	
Married women	Δ Total Testosterone	-18.32	0.98	-20.24	-16.39	<0.005
	Δ Free Testosterone	-0.35	0.03	-0.40	-0.30	<0.005
	Δ DHEA-S	-148.13	6.84	-161.59	-134.68	<0.005
	Δ HOMA-IR	-1.67	0.10	-1.87	-1.47	<0.005
	Δ Weight (263 Women)	-2.81	0.33	-3.48	-2.14	<0.005
	Δ Weight (151 Women in the obesity range)	-4.76	0.52	-5.79	-3.73	<0.005
	Δ BMI (263 Women)	-1.07	0.13	-1.32	-0.81	<0.005
	Δ BMI (151 Women in the obesity range)	-1.84	0.20	-2.23	-1.45	<0.005
	Δ mFG Score	-4.71	0.28	-5.25	-4.16	<0.005
Unmarried women	Δ Total Testosterone	-17.81	1.45	-20.68	-14.95	<0.005
	Δ Free Testosterone	-0.29	0.03	-0.35	-0.24	<0.005
	Δ DHEA-S	-67.22	9.63	-86.19	-48.25	<0.005
	Δ HOMA-IR	-0.95	0.11	-1.17	-0.73	<0.005
	Δ Weight (236 Women)	-2.11	0.31	-2.73	-1.50	<0.005
	Δ Weight (93 Women in the obesity range)	-5.71	0.47	-6.64	-4.78	<0.005
	Δ BMI (236 Women)	-0.87	0.13	-1.12	-0.61	<0.005
	Δ BMI (93 Women in the obesity range)	-2.36	0.19	-2.75	-1.98	<0.005
	Δ mFG Score	-4.85	.22	-5.28	-4.43	<0.005

**Abbreviations:** Δ, Delta Change; BMI, Body Mass Index; DHEA-S, Dehydroepiandrosterone Sulfate; HOMA-IR, Homeostatic Model Assessment for Insulin Resistance; mFG, Modified Ferriman-Gallwey; SE, Standard Error of Mean.

**Table 4:** The overall change in the domain score effect on the health-related quality of life using the PCOSQOL-47 for married women with polycystic ovary syndrome.

Domain Effect		PCOSQOL-47 Domains Scoring over 12 Months n (%)					
The Initial Effect of Enrollment	The Final Effect at the Study End	Psychological and Emotional Status Domain	Fertility and Sexual Life Domain	Body Image Domain	Hair Disorders and Acne Domain	Obesity and Menstrual Disorders Domain	Total
1 Marked	1 Marked	-	28 (10.6)	86 (32.7)	85 (32.3)	43 (16.3)	29 (11.0)
1 Marked	2 Marginal	14 (5.3)	3 (1.1)	87 (33.1)	127 (48.3)	119 (45.2)	170 (64.6)
1 Marked	3 Minimal	149 (56.7)	-	2 (0.8)	3 (1.1)	26 (9.9)	4 (1.5)
1 Marked	4 No effect	10 (3.8)	-	-	-	-	-
2 Marginal	2 Marginal	-	55 (20.9)	-	8 (3)	13 (4.9)	9 (3.4)
2 Marginal	3 Minimal	16 (6.1)	-	52 (19.8)	36 (13.7)	32 (12.2)	33 (12.5)
2 Marginal	4 No effect	74 (28.1)	-	-	-	-	-
2 Marginal	1 Marked	-	52 (19.8)	-	-	4 (1.5)	-
3 Minimal	3 Minimal	-	28 (10.6)	23 (8.7)	-	20 (7.6)	16 (6.1)
3 Minimal	4 No effect	-	-	13 (4.9)	-	-	-
3 Minimal	2 Marginal	-	29 (11)	-	-	1 (0.4)	-
3 Minimal	1 Marked	-	5 (1.9)	-	-	-	2 (0.8)
4 No effect	4 No effect	-	3 (1.1)	-	4 (1.5)	1 (0.4)	-
4 No effect	3 Minimal	-	51 (19.4)	-	-	3 (1.1)	-
4 No effect	2 Marginal	-	6 (2.3)	-	-	1 (0.4)	-
4 No effect	1 Marked	-	3 (1.1)	-	-	-	-

**Table 5:** The overall change in the domain score effect on the health-related quality of life using the PCOSQOL-42 for unmarried women with polycystic ovary syndrome.

Domain Effect		PCOSQOL-42 Domains Scoring over 12 Months n (%)					
The Initial Effect of Enrollment	The Final Effect at the Study End	Psychological and Emotional Status Domain	Menstrual Disorders and Fertility Domain	Body Image Domain	Hair Disorders and Acne Domain	Coping Domain	Total
1 Marked	1 Marked	4 (1.7)	2 (0.8)	2 (0.8)	33 (14)	54 (22.9)	4 (1.4)
1 Marked	2 Marginal	87 (36.9)	48 (20.3)	60 (25.4)	142 (60.2)	55 (23.3)	110 (46.6)
1 Marked	3 Minimal	54 (22.9)	101 (42.8)	90 (38.1)	12 (5.1)	27 (11.4)	45 (19.1)
1 Marked	4 No effect	-	1 (0.4)	3 (1.3)	-	-	-
2 Marginal	2 Marginal	2 (0.8)	3 (1.3)	-	6 (2.5)	-	-
2 Marginal	3 Minimal	65 (27.5)	31 (13.1)	37 (15.7)	39 (16.5)	36 (15.3)	62 (26.3)
2 Marginal	4 No effect	-	9 (3.8)	6 (2.5)	-	10 (4.2)	-
2 Marginal	1 Marked	-	20 (8.5)	-	-	-	-
3 Minimal	3 Minimal	15 (6.4)	-	14 (5.9)	4 (1.7)	14 (5.9)	15 (6.4)
3 Minimal	4 No effect	7 (3)	15 (6.4)	17 (7.2)	-	32 (13.6)	-
3 Minimal	2 Marginal	-	1 (0.4)	-	-	-	-
3 Minimal	1 Marked	-	-	-	-	-	-
4 No effect	4 No effect	2 (0.8)	4 (1.7)	7 (3.0)	-	8 (3.4)	-
4 No effect	3 Minimal	-	1 (0.4)	-	-	-	-
4 No effect	2 Marginal	-	-	-	-	-	-
4 No effect	1 Marked	-	-	-	-	-	-

**Table 6:** Summarized comparison of domain-specific HRQOL improvements between married and unmarried women with PCOS after one year of anti-androgen therapy.

Domain	Married Women (PCOSQOL-47)	Unmarried Women (PCOSQOL-42)	Key Observations
Psychological/Emotional	Highest improvement	Moderate improvement	More marked in married women
Fertility	Lowest improvement	High improvement	Greater relevance and perceived benefit in unmarried women
Sexual Function	Lowest improvement	Not assessed	Assessed only in PCOSQOL-47
Menstrual Irregularity	Significant improvement	Highest improvement	More significant in unmarried women
Hirsutism/Physical Signs	Significant improvement	Significant improvement	Similar magnitude of reduction in mFG score
Weight/BMI	Moderate improvement (−5%)	Moderate improvement (−5%)	Comparable across groups
Coping/Stress Handling	Moderate improvement	Lowest improvement	Coping showed least improvement in unmarried women
Overall HRQOL Score	Improved	Greater improvement	Unmarried women showed more overall gain

## Data Availability

The datasets generated and/or analyzed during the current study are not publicly available due to confidentiality, but are available upon reasonable request from the corresponding author.

## LIST OF ABBREVIATIONS

BMI: Body Mass Index
PCOS: Polycystic Ovary Syndrome
OCP: Oral Contraceptive Pills
FPG: Fasting Plasma Glucose
TT: Total Testosterone
DHEA-S: Dehydroepiandrosterone Sulfate
IR: Insulin Resistance

## STANDARDS OF REPORTING

STROBE guidelines were followed.
